# 270. New Onset Seizure Presented as Neurosyphilis

**DOI:** 10.1093/ofid/ofab466.472

**Published:** 2021-12-04

**Authors:** Shwe S Phyo, Cho T Zin, Zeyar Thet

**Affiliations:** 1 Kings County Infectious Disease, Brooklyn, New York; 2 Kings County Infectious Disease Clinic PC, Brooklyn, New York; 3 Wyckoff Heights Medical Center, Brooklyn, New York

## Abstract

**Background:**

The term “neurosyphilis” refers to infection of the central nervous system (CNS) by *Treponema pallidum*. It can occur at any time after initial infection. Early in the course of syphilis, the most common forms of neurosyphilis involve the cerebrospinal fluid (CSF), meninges, and vasculature (asymptomatic meningitis, symptomatic meningitis, and meningovascular disease). Late in disease, the most common forms involve the brain and spinal cord parenchyma (general paralysis of the insane and tabes dorsalis).

**Methods:**

A 31-year-old man who suddenly developed a new onset generalized tonic clonic seizure, was admitted to the emergency department. He had no history of epilepsy and denied any vision or gait problems. The brain MRI showed no abnormalities. He had a history of rapid plasma reagent (RPR) titer 1:32 and a positive fluorescent treponemal antibody absorption (FTA-ABS) test in 2017. However, the RPR result was non-reactive when he retested a week later and therefore was not diagnosed with syphilis and did not get treated at that time. His most recent RPR titer was 1:16. HIV serology and other STD tests were all negative. His wife and his 3 kids were negative for syphilis. Due to serological evidence of syphilis and neurological symptoms, we arranged him to get a lumbar puncture to rule out neurosyphilis.

**Results:**

His CSF study showed positive venereal disease research laboratory (VDRL), WBC cell count 44 cells/ul (lymphocytes 80%, Neutrophil 20%), Glucose 50 mg/dl, Protein 75 mg/dl. Based on the CSF study, he was diagnosed with neurosyphilis and was treated with intravenous Penicillin G 3-4 million units every 4 hours for 14 days, followed by Benzathine Penicillin 2.4million units intramuscularly on day 21.

**Conclusion:**

This is an unusual case because his false negative RPR result has hindered the prompt diagnosis and management of syphilis. RPR is a nontreponemal test and therefore it is not always reliable as a diagnostic criteria. False negatives in RPR may occur in certain conditions such as in early primary or in late stage syphilis and prozone phenomenon. This case illustrates the importance of using a reverse sequence algorithm in diagnosing syphilis. Thorough history taking is also crucial in conjunction with serological tests to determine the diagnosis and to ensure appropriate treatment.

**Disclosures:**

**All Authors**: No reported disclosures

